# Scar Sarcoidosis on ^18^F-FDG PET/CT

**DOI:** 10.22038/AOJNMB.2019.37888.1253

**Published:** 2019

**Authors:** Saurabh Arora, Nishikant Avinash Damle, Averilicia Passah, Animesh Ray, Manish Soneja, Sayantan Banerjee, Seema Kaushal, Manisha Jana, Madhavi Tripathi, Chandrasekhar Bal

**Affiliations:** 1 Department of Nuclear Medicine, All India Institute of Medical Sciences, New Delhi, India; 2 Department of Medicine, All India Institute of Medical Sciences, New Delhi, India; 3 Department of Pathology, All India Institute of Medical Sciences, New Delhi, India; 4 Department of Radiodiagnosis, All India Institute of Medical Sciences, New Delhi, India

**Keywords:** utaneous sarcoidosis, Scar sarcoidosis, ^ 18^F-FDG PET/CT

## Abstract

^18^F-labeled fluoro-2-deoxyglucose positron emission tomography/computed tomography (^18^F-FDG PET/CT) is an important imaging modality in the clinical workup of patients with chronic inflammatory disorders which present quite often with a fever of unknown origin. Sarcoidosis is a multisystem chronic inflammatory disorder with a wide clinical spectrum that can involve different organs. The diagnosis of sarcoidosis is usually based on the observation of noncaseating granulomas in biopsy specimens and exclusion of other granulomatous diseases. Skin involvement can occur in 20-25% of sarcoidosis cases. However, scar involvement in sarcoidosis is a rare condition. Herein, we present a case of multisystem sarcoidosis in a 45-year-old woman, who was previously treated with steroids and was in remission for 8 months. The patient presented with multiple skin nodules on the chest and back, a history of intermittent fever, headache, and mild itching at the abdominal scar site for 3 months. Blood investigations revealed elevated serum angiotensin-converting enzyme levels. The ^18^F-FDG PET/CT revealed a metabolically active involvement of the cutaneous tissue (posthysterectomy scar), apart from other sites of involvement. Biopsy of the scar site revealed multiple epithelioid cell granulomas with giant cells surrounding the collagenous fibers of the scar tissue.

## Introduction

Sarcoidosis is a multisystem granulomatous disease that is usually diagnosed based on biopsy specimens demonstrating noncaseating granulomas and excluding other granulomatous diseases (1). Skin involvement can occur in 20-25% of sarcoidosis cases (2). Nonetheless, scar sarcoidosis is a rare condition and accounts for 5.4-13.8% of cases with cutaneous sarcoidosis (3). Herein, we present a case of multisystem sarcoidosis in which ^18^F-FDG PET/CT revealed a metabolically active involvement of the cutaneous tissue (posthysterectomy scar). 

## Case report

Our case was a 45-year-old woman who was previously treated for sarcoidosis with steroids. The patient did not use steroids any longer and was in remission for 8 months. She presented with multiple skin nodules on the chest and back, a history of intermittent fever, headache, and mild itching at the abdominal scar site for 3 months. In addition, the patient had a history of laparotomy performed 20 years ago. 

**Figure 1:**
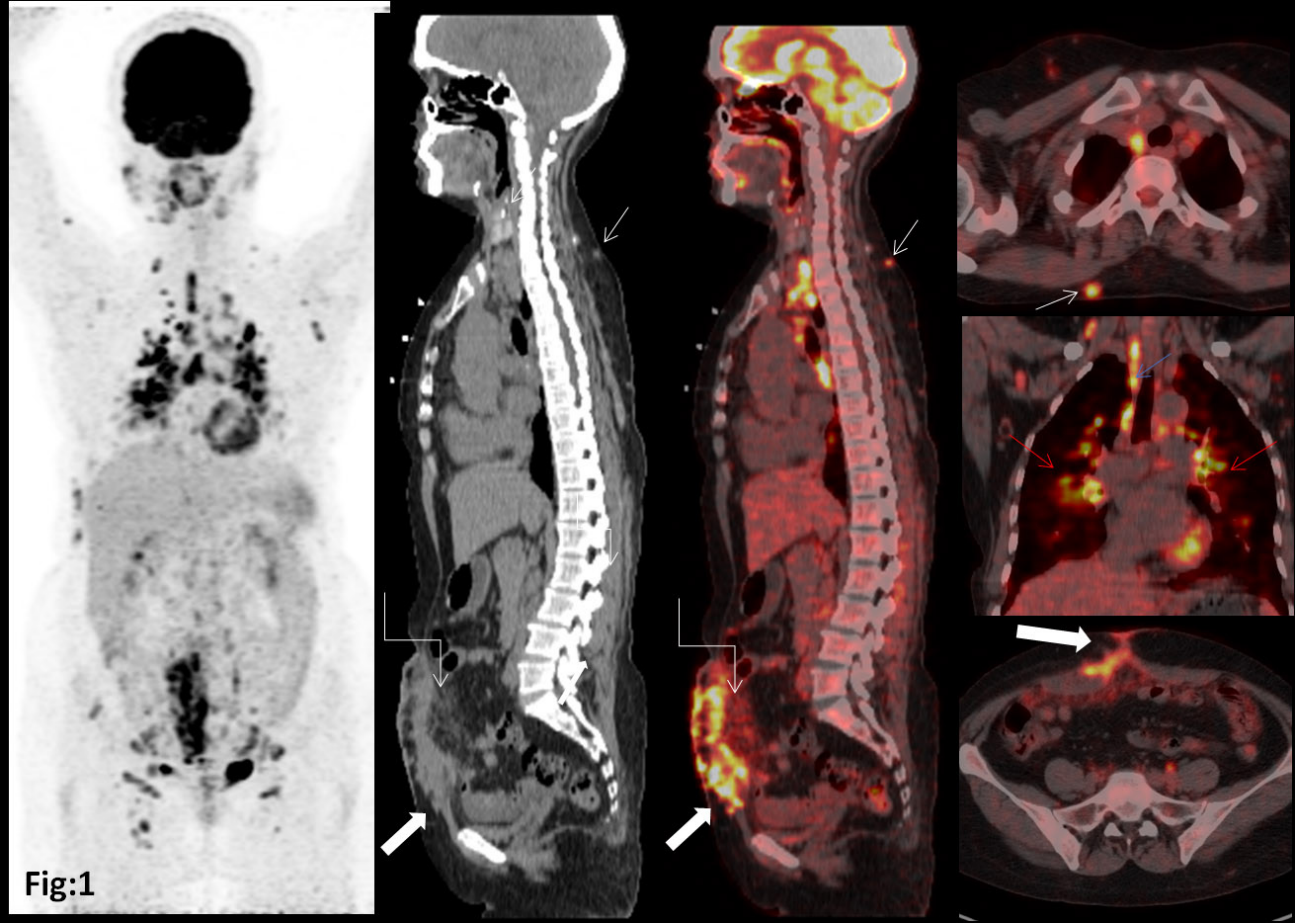
^18^F-FDG PET/CT show increased FDG uptake in multiple subcutaneous nodules (small white arrow), mediastinal nodes (blue arrow), and bilateral lung parenchymal involvement and anterior abdominal wall scar (thick white arrow) with the involvement of the omentum (curved white arrow)

**Figure 2:**
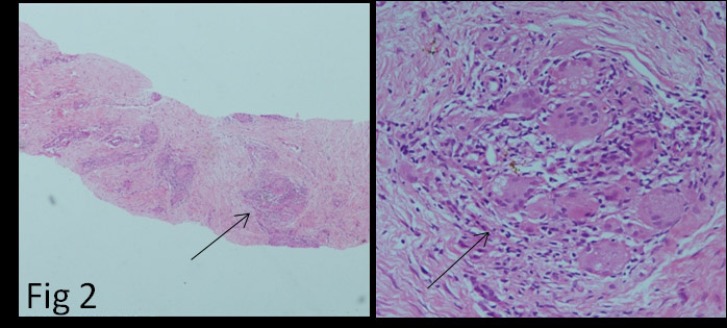
Microphotograph of the core biopsy of scar tissue (magnification X40 and X400) showing multiple epithelioid cell granulomas with giant cells

Blood examination revealed elevated serum angiotensin-converting enzyme (ACE) levels. The patient was suspected of disease relapse; therefore, she was subjected to ^18^F-FDG PET/CT to investigate her current disease status. The^ 18^F-FDG PET/CT demonstrated increased FDG uptake in multiple subcutaneous nodules (small white arrow), mediastinal nodes (blue arrow), bilateral lung parenchymal involvement (red arrows), and anterior abdominal wall scar (thick white arrow) with the involvement of the omentum (curved white arrow; Figure 1). 

To confirm the disease relapse and investigate the scar involvement, the patient was subjected to the biopsy of the scar site (i.e., metabolically active [FDG positive] site). Biopsy revealed multiple epithelioid cell granulomas with giant cells (black arrow) surrounding the collagenous fibers of scar tissue, thereby confirming the sarcoid infiltration of the scar site (Figure 2). 

## Discussion

Sarcoidosis is a multisystem chronic inflammatory disorder that has a wide clinical spectrum depending on the site of involvement. Cutaneous system involvement in sarcoidosis is a condition of low prevalence. There are few case reports regarding cutaneous scar involvement in sarcoidosis after trauma and surgery or vitiligo-associated scarring (4-6). 

The diagnosis of sarcoidosis is usually based on the histopathological evidence of noncaseating granulomas. However, the sampling of the internal organs is a difficult measure. On the other hand, for cases with skin involvement, the biopsy of the affected site can be accomplished easily (7). Similarly, in a previously published case, ^18^F-FDG PET/CT showed increased FDG uptake in multiple lymph nodes and a previous hysterectomy scar site, producing the scar sign suggesting sarcoidosis (8). 

In addition, in another study, ^18^F-FDG PET/CT demonstrated FDG uptake in a biopsy-proven scar sarcoidosis in a patient with a past history of adenocarcinoma colon and renal cell carcinoma (9). In the current study, ^18^F-FDG PET/CT revealed a metabolically active multisystem involvement affecting the cutaneous tissue in a patient with suspected sarcoidosis relapse.

In the current study, metabolic activity in the chronic scar was well correlated with the clinical involvement and facilitated the selection of the site for skin biopsy in a minimally invasive manner. The patient was started on steroid and responded well to the treatment. She showed a clinical improvement in scar itching within 3 days of initiating steroids. 

## Conclusion

The present study demonstrated the role of ^18^F-FDG PET/CT in assessing disease status in relapsed multisystem sarcoidosis. In this regard, ^18^F-FDG PET/CT facilitated the selection of the site of tissue sampling for confirming the diagnosis. The detection of an increased FDG uptake at the chronic scar sites in ^18^F-FDG PET/CT should be kept in mind as a differential diagnosis in appropriate clinical settings.
